# Preventing Establishment: An Inventory of Introduced Plants in Puerto Villamil, Isabela Island, Galapagos

**DOI:** 10.1371/journal.pone.0001042

**Published:** 2007-10-17

**Authors:** Anne Guézou, Paola Pozo, Christopher Buddenhagen

**Affiliations:** 1 Charles Darwin Research Station, Puerto Ayora, Galapagos, Ecuador; 2 Pacific Cooperative Studies Unit, University of Hawaii at Manoa, Honolulu, Hawai, United States of America; University of St. Andrews, United Kingdom

## Abstract

As part of an island-wide project to identify and eradicate potentially invasive plant species before they become established, a program of inventories is being carried out in the urban and agricultural zones of the four inhabited islands in Galapagos. This study reports the results of the inventory from Puerto Villamil, a coastal village representing the urban zone of Isabela Island. We visited all 1193 village properties to record the presence of the introduced plants. In addition, information was collected from half of the properties to determine evidence for potential invasiveness of the plant species. We recorded 261 vascular taxa, 13 of which were new records for Galapagos. Most of the species were intentionally grown (cultivated) (73.3%) and used principally as ornamentals. The most frequent taxa we encountered were *Cocos nucifera* (coconut tree) (22.1%) as a cultivated plant and *Paspalum vaginatum* (salt water couch) (13.2%) as a non cultivated plant. In addition 39 taxa were naturalized. On the basis of the invasiveness study, we recommend five species for eradication (*Abutilon dianthum, Datura inoxia, Datura metel, Senna alata* and *Solanum capsicoides*), one species for hybridization studies (*Opuntia ficus-indica*) and three species for control (*Furcraea hexapetala*, *Leucaena leucocephala* and *Paspalum vaginatum*).

## Introduction

Oceanic islands are particularly vulnerable to disturbance resulting from human activity [1] and more susceptible to invasion than continental ecosystems [2], [3]. This proves to be true in Galapagos where one of the major threats to the native plant communities comes from invasive introduced plants.

Located in the eastern Pacific Ocean, the Galapagos Islands straddle the equator, some 1000 kilometers west of the South American coast. The archipelago, of volcanic origin, is known to be one of the best preserved oceanic archipelagos in the world due to late arrival and establishment of man and is famous for the high level of endemism across all taxa–42% of the 553 native plant species are endemic. The latter group includes seven endemic genera including *Scalesia*, a famous example of adaptative radiation. So far only three native species have been reported as extinct [4], [5].

In spite of the fact that 95% of the archipelago is protected as a National Park, the persistence of the native plant communities and species are increasingly at risk, owing to direct and indirect pressures from the presence and activities of humans in the islands [6]. According to the IUCN criteria, 64% of the endemic flora is considered to be threatened [4], One of the most significant threats comes from introduced plant and animal species. Invasive plant species such as *Psidium guajava* (guava), *Cinchona pubescens* (quinine tree) and *Rubus niveus* (blackberry), and animal species such as the goat, pig and donkey have altered the composition and structure of natural ecosystems, and also have a significant negative impact on farming activities [7], [8]

The complete eradication or continuous control of established invasive and aggressive plants is difficult and, when possible, implies costly long-term programs [9], [10]. Many authors and invasion-specialists strongly recommend a prevention and early-detection strategy, removing species before they become established, and thus saving time and resources in the future, as well as preventing the impact these species would have on the natural systems [10]–[12].

In order to implement the early detection strategy, Randall [13] emphasizes the importance of creating a checklist of all exotic species already present to provide an accurate base-line.

Previous inventories of introduced plant species in Galapagos have not been carried out in a systematic, or exhaustive fashion, and given the importance of complete reliable information for carrying out an eradication campaign, a systematic program of inventories of the introduced plants of the urban and rural zones of Galapagos was initiated in 2002.

The present study is a component of this extensive program and focuses on the introduced plants of the urban part of Isabela Island, the village of Puerto Villamil. Puerto Villamil is the largest village on the island, and thus represents a focal point for the introduction of plant species. We registered the presence and distribution of all the introduced plant species in the village, and gathered information about their phenology, reproduction and state of naturalization. Every species recorded is assessed for its potential invasibility, and both sets of information are used to develop a list of priority species for complete eradication from Isabela Island. This inventory also contributed to the Charles Darwin Research Station (CDRS) Herbarium collection with new specimens of introduced plant species.

## Results and Discussion

We recorded a total of 261 vascular plant taxa in the village. All but two (identified only to genus) were identified to a species or subspecies level.

Up until the start of this study in September 2004, only 153 taxa of introduced plants had been reported for Isabela Island. Our study contributed 108 additional taxa, representing a 41.4% increase in the number of introduced taxa known from Isabela. This includes 13 new taxa for Galápagos (Table 1), and all except for *Abutilon dianthum* were being grown intentionally, mainly for ornamental use but also for medicinal purposes or as food. One of these species, *Lawsonia inermis* was determined to be fully naturalized in Puerto Villamil.

**Table 1:** Complete list of the introduced plant taxa encountered in Puerto Villamil.

Scientific name and Family	Common names English (Spanish)	Use	Growth form	Introduction status in Galapagos	Data for naturalisation assessmnent in Puerto Villamil
*Abutilon dianthum* (Malvaceae) 1,2			h	Es	notcu, sex1, fl, fr
*Acalypha amentacea wilkesiana* (Euphorbiaceae) 2	(Cresta de pavo)	orn	sh	Cu	cu, fl
*Acalypha hispida* (Euphorbiaceae) 2	(Cola de Zorro, Rabo de mono)	orn	sh	Cu	cu, fl
*Acalypha marginata* (Euphorbiaceae) 2		orn	sh	Cu	cu, fl
*Achillea millefolium* (Asteraceae) 2	Yarrow	orn	h	Es	Cu
*Achyranthes aspera* (Amaranthaceae)			h	Ac	notcu
*Agave americana* (Agavaceae) 2	Century Plant (Cabuya negra, Penco)	orn	s	Cu	cu
*Agave angustifolia* var. *marginata* (Agavaceae) 1, 2		orn	s	Cu	cu, asex2
*Aglaonema commutatum* (Araceae) 2		orn	h	Cu	Cu
*Allamanda cathartica* (Apocynaceae) 2	Yellowbell (Campana de oro, Copa de oro)	orn	v	Cu	cu, fl
*Allium cepa* (Alliaceae) 2	Onion, Spring onion (Cebolla paiteña, Cebolla blanca)	edi	h	Cu	Cu
*Allium sativum* (Alliaceae) 2	Garlic (Ajo)	edi	h	Cu	
*Allium schoenoprasum* (Alliaceae) 2	Chives (Cebollín)	edi	h	Cu	Cu
*Alocasia macrorrhizos* (Araceae) 2	(Camacho)	orn	h	Cu	cu, asex2
*Aloe arborescens* (Asphodelaceae) 2	(Sábila de Castilla)	orn	s	Cu	Cu
*Aloe cooperi* (Asphodelaceae) 1,2		orn	s	Cu	cu, asex2
*Aloe vera* (Asphodelaceae) 2	(Sabila)	med	s	Cu	cu, asex2, fl, fr
*Alternanthera tenella* (Amaranthaceae) 2	(Pata de paloma)	orn	h	Cu	Cu
*Amaranthus caudatus* (Amaranthaceae) 2	(Sangoreche verde, Quinoa)	med	h	Cu	cu, notcu, fl, fr
▸*Amaranthus dubius* (Amaranthaceae)			h	Ac	notcu, sex1, fl, fr
▸*Amaranthus lividus* (Amaranthaceae)			h	Ac	notcu, sex1, asex1, fl, fr
*Amaranthus spinosus* (Amaranthaceae)			h	Ac	notcu, fl, fr
*Ananas comosus* (Bromeliaceae) 2	Pineapple (Piña)	edi	s	Cu	Cu
*Annona cherimola* (Annonaceae) 2	Cherimoya (Chirimoya)	edi	sh	Es	
▸*Annona glabra* (Annonaceae)	Pond Apple (Anona)		t	NaQ	notcu, sex1, asex1, fl, fr
*Annona muricata* (Annonaceae)	Soursop (Guanábana)	edi	t	Es	cu, fr
*Antigonon leptopus* (Polygonaceae)	Coral Vine (Corazon bello)	orn	v	Es	cu, fl, fr
*Apium graveolens* (Apiaceae) 2	Celery (Apio)	edi	h	Cu	
*Aptenia cordifolia* (Aizoaceae) 2		orn	s	Cu	cu, asex2, fl, fr
*Asparagus densiflorus* (Asparagaceae) 2	Foxtail Fern	orn	v	Es	cu, fl, fr
*Asparagus setaceus* (Asparagaceae) 2	Asparagus Fern (Creston)	orn	v	Es	Cu
*Bauhinia monandra* (Caesalpiniaceae)	Orchid Tree (Orquidea del pobre)	orn	t	Cu	cu, notcu, sex2, fl, fr
*Beta vulgaris* var. *cicla* (Chenopodiaceae) 2	(Acelga)	edi	h	Cu	Cu
*Beta vulgaris* var. *vulgaris* (Chenopodiaceae) 2	Beetroot (Remolacha)	edi	h	Cu	
*Bidens pilosa* (Asteraceae)	(Amor seco)		h	NaQ	Notcu
*Blumea viscosa* (Asteraceae) 2			h	Ac	Notcu
*Borreria laevis* (Rubiaceae)			h	NaQ	notcu, sex1, fl, fr
*Bougainvillea* x *buttiana* (Nyctaginaceae) 2	(Bouganvilla, Veranera)	orn	sh	Cu	cu, fl
*Brasiliopuntia brasiliensis* (Cactaceae)		orn	s	Cu	Cu
*Brassica napus* (Brassicaceae) 2	(Nabo)	edi	h	Cu	
*Brassica oleracea* var. *botrytis* (Brassicaceae) 2	Cauliflower (Coliflor)	edi	h	Cu	cu, fl
*Brassica oleracea* var. *capitata* (Brassicaceae) 2	Cabbage (Col)	edi	h	Cu	cu, fl
*Brassica oleracea* var. *italica* (Brassicaceae) 2	Broccoli (Brócoli)	edi	h	Cu	cu, fl
*Brassica rapa* (Brassicaceae)			h	Es	notcu, fl, fr
*Breynia disticha* var. *nivosa* (Euphorbiaceae) 2	(Arbolito de navidad)	orn	sh	Cu	cu, sex2, asex2, fl, fr
*Brugmansia* x *candida* (Solanaceae) 2	(Floripondio, Guanto)	orn	sh	Es	cu, fl
*Bryophyllum daigremontianum* (Crassulaceae) 2		orn	s	Cu	cu, asex2
*Bryophyllum gastonis-bonnieri* (Crassulaceae) 2		orn	s	Cu	cu, sex2, asex2, fl, fr
▸*Bryophyllum pinnatum* (Crassulaceae) 2	Mother-of-Thousands (Hoja del aire)	orn-med	s	Es	cu, notcu, asex1, fl
*Bunchosia cornifolia* (Malpighiaceae) 2	Barbados Cherry (Cereza, Nicaragua)	orn	sh	Cu	cu, fr
▸*Caesalpinia bonduc* (Caesalpiniaceae)	(Mora)		sh	NaQ	notcu, sex1, fl, fr
*Caesalpinia pulcherrima* (Caesalpiniaceae) 2	Pride of Barbados	orn	sh	Es	cu, sex2, fl, fr
*Caladium bicolor* (Araceae) 2	Elephant's Ears (Corazón de Jesús)	orn	h	Cu	Cu
*Canna indica* (Cannaceae)	Indian Shot (Achira)	orn	h	Cu	cu, asex2
*Canna* x *generalis* (Cannaceae) 2	(Atzera, Platanillo)	orn	h	Es	cu, asex2, fl, fr
*Capsicum annuum* (Solanaceae) 2	Sweet Pepper (Aji, Pimiento)	edi	h	Cu	cu, fl, fr
*Capsicum frutescens* (Solanaceae)	Chili (Ají)	edi	h	Es	cu, fl, fr
*Caralluma hesperidum* (Asclepiadaceae) 1,2	(Pata de langosta)	orn	s	Cu	cu, asex2
▸*Carica papaya* (Caricaceae) 2	Pawpaw, Papaya (Papaya)	edi	t	Es	cu, notcu, sex1, sex2, fl, fr
▸*Catharanthus roseus* (Apocynaceae)	Madagascar Periwinkle (Chavelas)	orn	h	Es	cu, notcu, sex1, asex1, sex2, asex2, fl, fr
*Ceiba pentandra* (Bombacaceae)	Cotton Tree (Ceibo)	orn	t	Cu	cu, fr
*Celosia argentea argentea* (Amaranthaceae) 2	(Cresta de gallo, Rabo de conejo)	orn	h	Cu	cu, fl
*Celosia argentea cristata* (Amaranthaceae) 2	(Cresta de gallo)	orn	h	Cu	cu, sex2, asex2, fl, fr
▸*Cenchrus echinatus* (Poaceae)			h	Ac	notcu, sex1, asex1, fl, fr
*Centratherum punctatum* (Asteraceae) 2		orn	h	Es	cu, fl, fr
*Cereus peruvianus* var. 1 (Cactaceae) 2		orn	s	Cu	cu, asex2
*Cereus peruvianus* var. *monstrosus* (Cactaceae) 2		orn	s	Cu	Cu
▸*Chamaesyce ophthalmica* (Euphorbiaceae)			h	Ac	notcu, sex1, asex1, fl, fr
▸*Chenopodium ambrosioides* (Chenopodiaceae)	Wormseed, Mexican Tea (Paico)	med	h	Es	cu, notcu, sex1, sex2, fl, fr
▸*Chenopodium murale* (Chenopodiaceae)			h	Ac	notcu, sex1, fl, fr
*Chlorophytum comosum* (Anthericaceae) 2	(Mala madre)	orn	h	Cu	cu, fl
*Citrullus lanatus* (Cucurbitaceae)	Watermelon (sandía)	edi	v	Es	cu, notcu, fl, fr
*Citrus medica* (Rutaceae) 2	(Citrón)	edi	t	Cu	Cu
*Citrus reticul,ata* (Rutaceae) 2	Tangerine, Mandarin (Mandarina fina)	edi	t	Cu	Cu
*Citrus* x “limon-mandarina” (Rutaceae) 1,2	(Limón-mandarina)	edi	t	Cu	cu, fl, fr
*Citrus* x *aurantiifolia* (Rutaceae) 2	Lime (Limón, Limón verde)	edi	t	Es	cu, asex2, fl, fr
*Citrus* x *limon* (Rutaceae)	Lemon (Limón amarillo, Limón sútil)	edi	t	Es	cu, fl, fr
*Citrus* x *paradisi* (Rutaceae) 2	Grapefruit (Toronja)	edi	t	Cu	cu, notcu
*Citrus* x *sinensis* (Rutaceae) 2	Sweet Orange (Naranja dulce)	edi	t	Cu	Cu
*Clerodendrum thomsonae* (Verbenaceae) 2	Bleeding Hearts	orn	v	Cu	cu, fl
*Cocos nucifera* (Arecaceae) 2	(Coco)	edi-orn	t	Cu	cu, fl, fr
*Codiaeum variegatum* (Euphorbiaceae) 2	Croton (Espelma, Croton)	orn	sh	Cu	cu, fl
*Conyza bonariensis* (Asteraceae)			h	Ac	notcu, fl, fr
*Conyza canadensis* (Asteraceae)			h	Ac	notcu, fl
*Cordyline fruticosa* (Asteliaceae) 2		orn	sh	Cu	cu, asex2
*Coriandrum sativum* (Apiaceae) 2	Coriander (Cilantro)	edi	h	Cu	cu, fl, fr
*Coronopus didymus* (Brassicaceae)	Lesser Swine-cress (Mastuerzo)		h	Ac	notcu, sex1, fl, fr
*Crinum* x *amabile* var. *amabile* (Amaryllidaceae) 2	(Lirio de cinta)	orn	h	Cu	Cu
▸*Cucumis dipsaceus* (Cucurbitaceae)	(Huevo de tigre)		v	AcQ	notcu, sex1, fl, fr
*Cucumis melo* (Cucurbitaceae) 2	Melon (Melón)	edi	v	Cu	cu, fl, fr
*Cucumis sativus* (Cucurbitaceae) 2	Cucumber (Pepino)	edi	v	Cu	cu, fl
*Cucurbita moschata* (Cucurbitaceae) 2	Pumpkin, Winter Squash (Zapallo)	edi	v	Cu	cu, notcu, fl, fr
*Cucurbita pepo* (Cucurbitaceae) 2	Courgette, Marrow (Zucchini)	edi	v	Cu	Cu
*Cyanthillium cinereum* (Asteraceae) 2			h	AcQ	notcu, fl, fr
*Cymbopogon citratus* (Poaceae) 2	Lemon Grass (Hierba luisa)	med	h	Cu	cu, asex2
▸*Cynodon dactylon* (Poaceae)	Couch		h	Es	cu, notcu, sex1, asex1, sex2, asex2, fl, fr
*Cyperus odoratus* (Cyperaceae) 2			h	Ac	notcu, fl, fr
▸*Cyperus surinamensis* (Cyperaceae) 2	(Sombrilla)	orn	h	Es	cu, notcu, asex1, asex2, fl, fr
*Dactyloctenium aegyptium* (Poaceae) 2			h	Ac	notcu, asex1, fl, fr
*Dahlia pinnata* (Asteraceae) 2	Dahlia (Dalia)	orn	h	Cu	cu, fl, fr
*Datura inoxia* (Solanaceae) 2		orn	ssh	Es	notcu, fl, fr
▸*Datura metel* (Solanaceae) 2		orn	h	Cu	cu, notcu, sex1, sex2, fl, fr
*Daucus carota* (Apiaceae) 2	Carrot (Zanahoria)	edi	h	Cu	cu, fl
*Delonix regia* (Caesalpiniaceae) 2	Flame Tree, Flamboyant (Falsa acacia)	orn	t	Es	cu, notcu, sex1, sex2, fl, fr
*Dendranthema* x *grandiflorum* cv.'Peggy Stevens' (Asteraceae) 2	Chrysanthemum (Pomo)	orn	h	Cu	cu, fl
*Dieffenbachia picta* (Araceae) 2	Dumb Cane (Millonaria, Chucha)	orn	h	Cu	Cu
*Dieffenbachia seguine* (Araceae) 2		orn	h	Cu	cu, asex2
*Digitaria ciliaris* (Poaceae)			h	NaQ	sex1
*Digitaria horizontalis* (Poaceae)			h	Ac	notcu, fl
▸*Digitaria setigera* (Poaceae)			h	Ac	notcu, sex1, fl, fr
*Dracaena angustifolia* (Dracaenaceae) 2		orn	ssh	Cu	
*Dracaena deremensis* (Dracaenaceae) 2		orn	ssh	Cu	Cu
*Dracaena fragrans* (Dracaenaceae) 2		orn	ssh	Cu	Cu
*Dypsis lutescens* (Arecaceae) 2	Golden Cane Palm (Palma enana)	orn	t	Cu	Cu
*Echeveria peacockii* (Crassulaceae) 1,2		orn	s	Cu	Cu
*Echinopsis eyriesii* (Cactaceae) 2		orn	s	Cu	Cu
*Echinopsis pachanoi* (Cactaceae) 2		orn	s	Cu	
▸*Eleocharis geniculata* (Cyperaceae)			h	NaQ	notcu, sex1, asex1, fl, fr
▸*Eleusine indica* (Poaceae)			h	Ac	notcu, sex1, asex1, fl, fr
*Eleutherine bulbosa* (Iridaceae) 2	(Vara de justicia)	orn	h	Cu	cu, asex2
*Epiphyllum oxypetalum* (Cactaceae) 2	(Galan de noche)	orn	s	Cu	Cu
*Epipremnum pinnatum* (Araceae) 2	(Enredadera, Cortina)	orn	v	Cu	cu
▸*Eragrostis amabilis* (Poaceae) 2			h	Ac	notcu, sex1, fl
*Erythrina smithiana* (Fabaceae) 2	(Porotillo)	oth	t	Cu	Cu
*Eucharis* x *grandiflora* (Amaryllidaceae) 2		orn	h	Cu	Cu
*Eucrosia bicolor* (Amaryllidaceae) 2		orn	h	Cu	notcu, fl
▸*Euphorbia cyathophora* (Euphorbiaceae)		orn	h	Es	cu, notcu, sex1, sex2, fl, fr
▸*Euphorbia graminea* (Euphorbiaceae)	Grassleaf Spurge		h	Ac	notcu, sex1, fl, fr
*Euphorbia heterophylla* (Euphorbiaceae) 2	Mexican Fire Plant, Wild Poinsettia		h	Es	
*Euphorbia lactea* (Euphorbiaceae) 2		orn	ssh	Cu	Cu
*Euphorbia milii* (Euphorbiaceae) 2	Crown of Thorns (Corona del Señor, Corona de Cristo)	orn	ssh	Cu	Cu
*Euphorbia pulcherrima* (Euphorbiaceae) 2	Poinsettia (Flor de Pascua/Panamá)	orn	sh	Es	Cu
*Ficus benjamina* (Moraceae) 2	(Ficus)	orn	t	Cu	Cu
*Ficus carica* (Moraceae) 2	Edible Fig (Higo)	edi	sh	Cu	cu, fl, fr
*Ficus elastica* (Moraceae) 2	(Caucho)	orn	t	Cu	Cu
▸*Furcraea hexapetala* (Agavaceae)	(Cabuya, Penco blanco)	orn	s	Es	cu, sex2, fr
*Gazania rigens* (Asteraceae)		orn	h	Cu	cu, fl
*Glechoma hederacea* (Lamiaceae) 1,2	(Alivia dolor, Cura todo)	med	h	Es	cu, asex2, fl
*Gliricidia sepium* (Fabaceae)	(Mata Raton, Madero Negro, Nacedero)	oth	t	Cu	cu, notcu, sex2, fl, fr
*Haworthia attenuata* (Asphodelaceae) 2		orn	s	Cu	cu, asex2, fl
*Helianthus annuus* (Asteraceae)	Sunflower (Girasol)	orn	ssh	Cu	cu, fl, fr
*Hemerocallis* hybrids (Liliaceae) 2	(Espiga de San Antonio)	orn	h	Cu	
*Hibiscus radiatus* (Malvaceae) 2	Monarch Rosemallow (Amapola)	orn	h	Es	cu, notcu, sex1, sex2, asex2, fl, fr
*Hibiscus rosa-sinensis* (Malvaceae) 2	(Peregrina)	orn	sh	Es	cu, fl, fr
*Hibiscus rosa-sinensis* var. *schizopetalus* (Malvaceae) 2	(Peregrino)	orn	sh	Cu	cu, fl
*Hippeastrum puniceum* (Amaryllidaceae) 2	Amaryllis	orn	h	Cu	cu, asex2
*Hippeastrum reticulatum* (Amaryllidaceae) 2		orn	h	Cu	Cu
*Hippeastrum* x Dutch hybrids (Amaryllidaceae) 1,2	Amaryllis (Amarilis)	orn	h	Cu	cu, fl
*Hoya carnosa* (Asclepiadaceae) 2	(Hoja de cera)	orn	s	Cu	
*Huernia aspera* (Asclepiadaceae) 2		orn	s	Cu	Cu
*Hydrangea macrophylla* (Hydrangeaceae)	Hydrangea (Hortensia)	orn	ssh	Cu	cu, fl
*Hylocereus polyrhizus* (Cactaceae) 2		orn	ssh	Es	cu, asex2, fl
*Hymenocallis pedalis* (Amaryllidaceae) 2	Spider Lily	orn	h	Cu	cu, notcu
*Inga edulis* (Mimosaceae) 2	(Guava (bejuco), Guava de mico)	edi	t	Es	cu, fl, fr
*Ipomoea batatas* (Convolvulaceae)	Sweet Potato (Camote)	edi	v	Es	cu, asex2, fl
*Ipomoea quamoclit* (Convolvulaceae) 2		orn	v	Cu	
*Jatropha curcas* (Euphorbiaceae) 2	(Piñon)	oth	sh	Es	cu, fl, fr
*Kalanchoe blossfeldiana* (Crassulaceae) 2		orn	s	Cu	cu, asex2, fl, fr
*Kalanchoe fedtschenkoi* (Crassulaceae) 2		orn	s	Cu	
*Kalanchoe tubiflora* (Crassulaceae) 2		orn	s	Es	cu, asex2
▸*Lawsonia inermis* (Lythraceae) 1,2	Henna (Reseda)	orn	h	Cu	cu, notcu, sex1, fl, fr
*Leucaena leucocephala* ssp. *glabrata* (Mimosaceae) 2	(Ipel ipel)		sh	Es	cu, fl
*Malpighia emarginata* (Malpighiaceae) 2		edi-orn	t	Cu	cu, fl
▸*Malvastrum coromandelianum* (Malvaceae)			h	Ac	notcu, sex1, fl, fr
*Malvaviscus arboreus* (Malvaceae) 2		orn	sh	Cu	cu, fl, fr
*Mangifera indica* (Anacardiaceae) 2	Mango (Mango)	edi	t	Es	cu, fl, fr
*Manihot esculenta* (Euphorbiaceae) 2	Cassava, Manioc (Yuca)	edi	sh	Cu	Cu
*Matricaria recutita* (Asteraceae) 2	Chamomile (Manzanilla)	med	h	Cu	cu, fl
*Matthiola incana* (Brassicaceae) 1,2	Stock	orn	h	Cu	cu, notcu, sex2, asex2, fl, fr
*Medicago sativa* (Fabaceae) 2	Alfalfa, Lucerne (Alfalfa)	med	h	Cu	
*Melia azedarach* (Meliaceae)	Chinaberry, Persian Lilac (Jasmin de Arabia, San Jacinto)	orn	t	Es	cu, fl, fr
*Mentha* x *piperita* (Lamiaceae)	Peppermint (Menta, Hierbabuena)	med	h	Es	cu, asex2, fl, fr
*Mirabilis jalapa* (Nyctaginaceae)	Marvel of Peru, Four O'Clock Plant (Buenas tardes)	orn	h	Es	notcu, fl, fr
*Musa acuminata* “Guineo” (Musaceae) 2	Banana (Guineo)	edi	h	Cu	cu, asex2
*Neomarica gracilis* (Iridaceae) 2		orn	h	Cu	cu, fl
*Nephrolepis cordifolia cordifolia* (Davalliaceae)		orn	h	Cu	cu, asex2
*Nephrolepis exaltata* cv. Gretnae (Davalliaceae) 2		orn	h	Cu	cu, asex2
*Nephrolepis exaltata* cv. Norwoodii (Davalliaceae) 2		orn	h	Cu	Cu
*Nerium oleander* (Apocynaceae)	Oleander (Laurel)	orn	sh	Cu	cu, fl, fr
*Ocimum basilicum* var. *basilicum* (Lamiaceae) 2	Basil (Albaca)	edi	h	Cu	cu, fl, fr
*Odontonema cuspidatum* (Acanthaceae) 2		orn	sh	Es	cu, fl
*Opuntia* cf. *monacantha* ssp. *monacantha* (Cactaceae) 2		orn	s	Cu	Cu
*Opuntia* cf. *monacantha* var. *variegata* (Cactaceae) 2		orn	s	Cu	Cu
*Opuntia dillenii* (Cactaceae) 2		orn	s	Cu	Cu
*Opuntia ficus-indica* (Cactaceae) 2	Prickly Pear	orn	s	Es	Cu
*Opuntia microdasys* var. 1 (Cactaceae) 2		orn	s	Cu	Cu
*Origanum vulgare* (Lamiaceae) 2	Orégano (Oregano)	edi	h	Cu	cu, asex2
*Oxalis corniculata* (Oxalidaceae)			h	AcQ	notcu, sex1, asex1, fl, fr
▸*Paspalum vaginatum* (Poaceae) 2	Saltwater Couch		h	Na	notcu, sex1, asex1, fl, fr
▸*Passiflora edulis* (Passifloraceae)	Maracuya (Maracuyá)	edi	v	Es	cu, notcu, sex1, fl
*Pedilanthus tithymaloides* (Euphorbiaceae) 2	(Zapatillas rojas)	orn	ssh	Cu	cu, asex2, fl
*Pelargonium graveolens* (Geraniaceae) 2	(Malva olorosa, Esencia de rosa)	orn-med	h	Cu	Cu
*Pelargonium* x *hortorum* (Geraniaceae) 2	Geranium (Geranio)	orn	h	Cu	cu, fl, fr
*Pennisetum purpureum* (Poaceae)	Elephant Grass (Pasto elefante)		h	Es	Cu
*Peperomia* sp. 2 (Piperaceae) 1,2		orn	h	Cu	Cu
*Petroselinum crispum* (Apiaceae) 2	Parsley (Perejil)	edi	h	Es	Cu
*Phaseolus lunatus* (Fabaceae) 2	Butter Bean, Lima Bean (Habichuela)	edi	v	Cu	cu, fl, fr
*Phaseolus vulgaris* (Fabaceae) 2	(Fréjol, Vainita)	edi	v	Cu	Cu
*Phoenix dactylifera* (Arecaceae)	Date Palm (Dátil)	orn	t	Cu	Cu
*Phyllanthus acidus* (Euphorbiaceae) 2	(Grosella)	edi	t	Cu	cu, sex2, fl, fr
▸*Plantago major* (Plantaginaceae)	Greater Plantain (Llanten)	med	h	Ac	cu, notcu, sex1, asex1, sex2, fl, fr
*Plectranthus unguentarius* (Lamiaceae) 2	(Oreganón, orégano)	edi	h	Cu	cu, asex2
*Polyscias guilfoylei* (Araliaceae) 2		orn	sh	Cu	Cu
*Polyscias scutellaria* (Araliaceae) 2		orn	sh	Cu	Cu
▸*Porophyllum ruderale* ssp. *macrocephalum* (Asteraceae)	(Ruda gallinazo)		h	Ac	notcu, sex1, fl, fr
*Portulaca grandiflora* (Portulacaceae) 2	(Flor de un día)	orn	s	Cu	cu, asex2, fl, fr
▸*Portulaca oleracea* (Portulacaceae)	Purslane (Verdolaga)	med	s	NaQ	notcu, sex1, asex1, fl, fr
*Portulaca pilosa* (Portulacaceae) 2		orn	s	Cu	cu, sex2, asex2, fl, fr
*Portulaca umbraticola* (Portulacaceae) 2		orn	s	Na	cu, asex2, fl, fr
*Pritchardia lanigera* (Arecaceae) 2		orn	t	Cu	Cu
*Priva lappulacea* (Verbenaceae)	(Cadillo)		h	Ac	notcu, sex1, fl, fr
*Pseuderanthemum carruthersii* (Acanthaceae) 2		orn	sh	Cu	cu, fl
*Psidium guajava* (Myrtaceae)	Guava (Guayabo)	edi	t	Es	cu, notcu, fr
*Ptychosperma elegans* (Arecaceae) 2		orn	t	Cu	cu, fl, fr
*Punica granatum* (Punicaceae)	Pomegranate (Granada)	edi	sh	Cu	cu, fl, fr
*Raphanus sativus* (Brassicaceae)	Radish (Rábano)	edi	h	Es	cu, fl, fr
▸*Ricinus communis* (Euphorbiaceae) 2	Castor Oil (Higuerrilla)		ssh	Es	notcu, sex1, fl, fr
*Rosa* hybrid cul,tivars (Rosaceae) 2	Rose (Rosa)	orn	ssh	Cu	cu, fl
*Roystonea regia* (Arecaceae) 2	Royal Palm (Palma real)	orn	t	Cu	
*Ruellia malacosperma* (Acanthaceae) 2		orn	h	Es	cu, notcu, sex2, asex2, fl, fr
*Russelia equisetiformis* (Scrophulariaceae) 2	(Lluvia de fuego, Lluvia de coral)	orn	sh	Cu	cu, fl, fr
*Ruta graveolens* (Rutaceae) 2	Rue (Ruda)	med	ssh	Cu	cu,
*Saccharum officinarum* (Poaceae) 2	Sugarcane (Caña de azucar)	edi	h	Cu	cu, asex2
*Sansevieria trifasciata* (Dracaenaceae) 2	Mother-in-law's Tongue (Lengua de suegra)	orn	h	Es	cu, asex2, fl
*Schefflera arboricola* (Araliaceae) 2	(Cheflera)	orn	ssh	Cu	cu,
▸*Senna alata* (Caesalpiniaceae)	(Palo de abejón)	orn	ssh	Es	cu, notcu, sex1, fl, fr
*Senna obtusifolia* (Caesalpiniaceae)	(Dormidera)		h	Ac	notcu,
▸*Sida acuta* (Malvaceae)	(Escoba negra)		h	Ac	notcu, sex2, fl
▸*Sida ciliaris* (Malvaceae) 2			h	Ac	notcu, sex1, fl, fr
▸*Sida rhombifolia* (Malvaceae)	(Escobilla)		h	Ac	notcu, sex1, fl, fr
▸*Solanum americanum* (Solanaceae)	Black Nightshade (Hierba mora)	med	h	NaQ	notcu, sex1, fl, fr
*Solanum betaceum* (Solanaceae) 2	Tree Tomato, Tomatillo (Tomate de arbol)	edi	sh	Cu	
*Solanum capsicoides* (Solanaceae) 1,2		orn	h	Cu	cu, notcu, fl, fr
*Solanum lycopersicum* (Solanaceae)	Tomato (Tomate riñon)	edi	h	Es	cu, notcu, sex1, sex2, asex2, fl, fr
*Solanum melongena* (Solanaceae) 2	Aubergine, Garden Egg, Eggplant (Berenjena)	edi	sh	Cu	
*Solanum pimpinellifolium* (Solanaceae) 2			h	Es	notcu, sex1, fl, fr
*Solanum quitoense* (Solanaceae) 2	Lulo (Naranjilla)	edi	sh	Es	
*Solanum tuberosum* (Solanaceae) 2	Potato (Papa)	edi	h	Cu	cu, notcu
*Solenostemon scutellarioides* (Lamiaceae) 2	(Coleos)	orn	h	Cu	Notcu
*Spinacia oleracea* (Chenopodiaceae) 2	Spinach (Espinaca)	edi	h	Cu	Cu
*Spondias purpurea* (Anacardiaceae)	(Ciruelo)	edi	t	Es	cu, fl, fr
*Swietenia macrophylla* (Meliaceae) 2	Mahogany (Caoba)	oth	t	Cu	cu, fl, fr
▸*Synedrella nodiflora* (Asteraceae)			h	Ac	notcu, sex1, fl, fr
*Syngonium podophyllum* (Araceae) 2		orn	v	Cu	Cu
*Tagetes erecta* (Asteraceae) 2		orn	h	Cu	cu, fl, fr
*Tamarindus indica* (Caesalpiniaceae)	Tamarind (Tamarindo)	edi	t	Cu	cu, fl, fr
*Taraxacum officinale* (Asteraceae) 2	Dandelion (Taraxaco, Diente de león)	med	h	AcQ	Cu
*Terminalia catappa* (Combretaceae)	Indian Almond (Almendro)	orn	t	Cu	cu, sex2, fl, fr
*Thuja orientalis* (Cupressaceae) 2	(Ciprés)	orn	t	Cu	
*Tradescantia* sp. 2 (Commelinaceae) 2		orn	h	Cu	cu, asex2
▸*Tribulus cistoides* (Zygophyllaceae)	(Cacho de chivo)		v	NaQ	notcu, sex1, asex1, fl, fr
*Tribulus terrestris* (Zygophyllaceae)	(Cacho de chivo)		v	NaQ	notcu, fl, fr
*Tropaeolum majus* (Tropaeolaceae) 2	Nasturtium (Mastuerzo)	med	v	Cu	cu, fl
*Vigna unguicul,ata* (Fabaceae) 2	Cowpea, Black-eyed Bean (Verdura, Fréjol chileno)	edi	v	Cu	cu, fl, fr
*Vitis vinifera* (Vitaceae) 2	Grape (Uva)	edi	v	Cu	Cu
*Xanthosoma sagittifolium* (Araceae) 2	(Otoy)	orn	h	Es	cu, asex2
*Yucca guatemalensis* (Agavaceae) 2	(Flor de novia, Peine de indio)	orn	sh	Cu	cu, asex2, fl
*Zantedeschia aethiopica* (Araceae) 1,2	(Cartucho)	orn	h	Cu	
*Zea mays* (Poaceae) 2	Maize (Mais)	edi	h	Cu	cu, fl
*Zephyranthes rosea* var. *candida* (Amaryllidaceae) 2		orn	h	Cu	cu, asex2, fl, fr
▸*Zoysia matrella* var. *pacifica* (Poaceae)	(Césped chino)	orn	h	Es	cu, notcu, sex1, asex1, sex2, asex2, fl, fr

Legend : Growth form: t) tree; sh) shrub; ssh) subshrub; h) herb; v) vine. Introduction status in Galapagos: Na) Native (not endemic); NaQ) Doubtfully native, possibly introduced; Cu) Cultivated (introduced for culture, not naturalized); Es) Escaped (introduced for culture, naturalized); Ac) Accidental (introduced unintentionnally, naturalized); AcQ) Doubtfully accidental (introduced, naturalized but it is not known if introduction was casual or intentional). Naturalization assessment: cu) cultivated; notcu) not cultivated; sex1) sexual regeneration from a non cultivated plant; sex2) sexual regeneration from a cultivated plant; asex1) asexual regeneration from a non cultivated plant; asex2) asexual regeneration from a cultivated plant; fl) presence of flowers; fr) presence of fruits; ▸fully naturalized taxon in Puerto Villamil.

1 indicates a new record for Galapagos,

2 indicates a new record for Isabela Island

The five most frequently recorded species were *Cocos nucifera (*coconut tree) (22.1%), *Paspalum vaginatum* (salt water couch) (13.2%), *Portulaca oleracea* (common purslane) (12.7%), *Aloe vera* (aloe) and *Annona glabra* (pond alligator apple) (12.2%). *C. nucifera* and *A. vera* had been planted intentionally (cultivated) and were used for food and medicine respectively; the other three species were growing in the properties without having been planted (not cultivated) and were not given any use.

### Distribution of species

**Figure 1:**
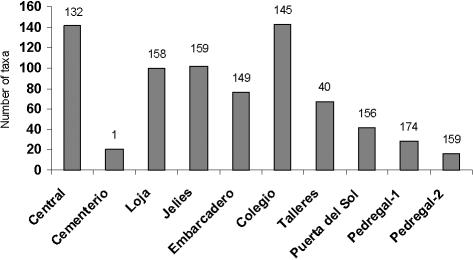
Total number of introduced plant taxa in each neighbourhood in Puerto Villamil. Numbers above the bar indicate the number of properties surveyed in each neigbourhood. The neighbourhoods are organized by time since establishment with oldest neighborhoods on the left.

The number of taxa recorded in each neighborhood reflected the dynamics of colonization as well as the activity of the dwellers (Figure 1). The neighborhoods with the highest numbers of taxa were the older ones and/or the ones containing the hotels, restaurants and shops of the village, such as *Central* and *Colegio*. The exception was the cemetery, which although it was established early, is a single property with a very different use from the other neighbourhoods.

Almost half (43.2%) of the selected properties had no introduced plants present, most of these occurring in neighbourhoods that have been established in the last 5 years. The neighborhoods of *Pedregal* 1 and *Pedregal* 2 were created in 2001 and 2003 respectively; others, such as *Embarcadero* and *Puerta del Sol*, though older, had only recently started a process of complete urbanization. These four neighborhoods still contain unaltered natural zones such as lava fields and mangroves, and include many vacant lots which are still covered in native vegetation.

### Uses and habits

The majority of the introduced plants in Puerto Villamil (82% of all taxa) were given a use. The principal use was ornamental (50% of all taxa), followed by food (22%), medicinal (5%) and mixed uses (5%) such as shade or fence plants in addition to the previous uses.

The most frequent growth form was the herbaceous form (48% of all taxa) followed by tree and succulent (each one 13%), shrub (12%), vine (9%) and subshrub (6%) (Table 1).

### Naturalization analysis

Of 236 taxa analysed, 167 taxa (70.8%) were being cultivated and 52 taxa (22.0%) not cultivated. The remaining 17 taxa (7.2%) were both cultivated and not cultivated. Forty four ornamental taxa were found to be regenerating without human assistance.

An analysis of naturalization identified 39 taxa as being completely naturalized in Puerto Villamil, with additional taxa in initial phases of naturalization.

### Invasive species

Although we did not detect any species displaying invasive behavior within the urban perimeter, it revealed three other important groups of plants:

- Species that have recently started to display an invasive behavior in the village but for which eradication will be difficult as their growth form prevents easy identification (e.g. species within the family Poaceae), or the plants are of importance to their owners as an ornamental. This group includes *Cynodon dactylon, Dactyloctenium aegyptium, Hylocereus polyrhizus, Ruellia malacosperma* and *Zoysia matrella* var. *pacifica*,
- Species with a limited distribution in the village but which are already recognized as aggressive invaders in nearby areas of the Galapagos National Park or in the rural zone Isabela, making the feasibility of their eradication from the Island difficult. This group includes *Paspalum vaginatum* (previously misidentified as *Pennisetum clandestinum* or kikuyo), *Leucaena leucocephala* var. *glabrata* and *Furcraea hexapetala*. *P. vaginatum* stands out as a special case: it is a native species that behaves aggressively in disturbed as well as in natural zones of Southern Isabela. In some areas of wetlands, outside of the urban perimeter, it appears to dominate the landscape, affecting water-movement, and soil moisture content [14].
- Species with a limited distribution in Isabela but that are recorded from elsewhere as being aggressive, and thus should be prioritized for eradication. This last group includes six species that we recommend as candidates for eradication:

***Abutilon diantum*** K. Presl (Syn. *A. sylvaticum, laxum*) is a subshrub from Perú [15]. Two congenerics are considered weeds in natural areas: *A. grandifolium* is invasive in Hawaii and *A. theophrasti* in four states of the USA [16]. In Galapagos, *A. dianthum* was found for the first time in Puerto Villamil (2004), in one property and is thus considered eradicable.

***Datura inoxia*** Mill. (Syn. *D. guayaquilensis*, *meteloides*), downy thornapple, is an herb from Mexico [17]. In South Africa, *D. inoxia* is categorized as a prohibited weed to be controlled in all situations [18]. In Western Australia, it escaped from yards and is now a weed of disturbed areas [17]. In Ecuador, this introduced cultivated species is in coastal regions as well as in the Sierra [19]. The first and only record for Galapagos is from Puerto Villamil (2004) where it is considered an introduced escapee. This species was found in a single property.

***Datura metel*** L. (Syn. *D. alba*), angel's trumpet or horn of plenty, is a shrub native to the Americas. In Western Australia, it is considered as a garden escapee and has naturalized in disturbed areas [17]. In Hawai'i, Fiji and other islands this species is occasionally cultivated and sparingly naturalized at low elevations in open, dry, disturbed areas, in waste places and sandy beaches [20]. In Galápagos, this introduced, cultivated species was first recorded in Puerto Ayora on Santa Cruz Island in 2002 (CDRS unpublished data). In 2004, we recorded *D. metel* as an escapee in five properties of Puerto Villamil.

***Senna alata*** (L.) Roxburgh (Syn. *Cassia alata*), candle stick tree is a native shrub of tropical America (Mexico). It is declared noxious in Australia (Northern Territory) where it grows in perennially moist areas, and occasionally on disturbed and overgrazed areas. In Hawai'i, it is considered poisonous for livestock and fish, and when cultivated as an ornamental, it attracts scale insects, thrips, and grasshoppers [20], [21]. In Galapagos, this introduced escapee was first recorded in 1986, cultivated in a farm of San Cristóbal Island. In 2004, it was detected on 16 properties of Puerto Villamil as well as in one farm of Isabela Island (CDRS unpublished data).

***Solanum capsicoides*** Allioni (Syn. *S. ciliatum*), cockroach berry, a native herb of coastal Brazil is now widely distributed as a weed in the tropical regions of the world. All parts of the plant are poisonous to livestock [21]. The Institute of Pacific Islands Forestry [20] recommends this species for control and eradication. In Galápagos, it was first recorded in 2004 as an introduced, cultivated species, in five properties the village and five farms of Isabela Island (CDRS unpublished data). In Puerto Villamil, the plant known as “naranjilla silvestre”, was often cultivated and has been reported as escaped in the neighbouring San Cristobal Island.

***Opuntia ficus-indica*** (L.) P. Miller (Syn. *Cactus ficus-indica; O. engelmanii, megacantha or tuna*), prickly-pear is a native tree or shrub from Mexico [22]. The WWF included this species in a National list of naturalized invasive and potentially invasive garden plants [23]. In Ecuador, this introduced plant grows wild in coastal areas and the Sierra region [19]. Significant genetic diversity within the genus *Opuntia* and natural occurrence of interspecific Natural and artificial *Opuntia* hybrids have been reported, [24]. *O*. *ficus-indica* was first recorded as an introduced escapee in Galápagos in 1991 on San Cristóbal Island. In Santa Cruz, it was found cultivated as an ornamental in six properties (CDRS unpublished data), while in Isabela, we only found it once in Puerto Villamil. The hybridization capacity of *O. ficus-indica* suggests the possibility of hybridization with endemic *Opuntia* taxa. We therefore recommend specific hybridization studies in order to orient management actions for *O. ficus-indica* and/or any other introduced *Opuntia* species in Galapagos.

### Conclusion

There are four steps in the battle against introduced species: prevention, early detection, eradication and control [25]. The results of the inventory reported in this paper are an important weapon in this battle: providing a baseline to allow detection of new introductions, and identifying species with a limited distribution that have the potential to become invasive. Removal of all individuals of each species at this early stage of the invasive process is a cost-effective approach to avoiding other significant invasions of the natural habitat in the long term.

## Materials and Methods

The field work was carried out during five weeks, between October 2004 and April 2005 and a last visit in September 2005.

All properties/lots in Puerto Villamil (1193 lots) were visited, 597 of them for complete inventory (selected lots) and 596 only for detection of additional species (non-selected lots).

### Study area

#### Geographical

Located on the western side of the Galápagos, Isabela is the largest island of the archipelago. The village of Puerto Villamil is situated along the southern coast, on a wide east-west barrier beach, met to the north by fields of basaltic lava from Sierra Negra Volcano. The semi-arid, subtropical climate is dominated by cold ocean currents driven by southeast trade winds, with two seasons, a dry and cool season from June to December and a wet and warm season from January to June. The overall variation of the air temperature is low. In Puerto Villamil, means ranging from 19 to 26°C have been registered during the cool season and from 22 to 30°C in the warm season. Precipitation averages 11 mm per month in the garua season and 48 mm per month in the hot season, except in El Nino years where the rainfall is much higher.

#### Social and historical

Even though Isabela was visited earlier it was truly colonized in 1897 by Antonio Gil who founded the village of Puerto Villamil, and then established a farm in the humid zone, on the slope of Sierra Negra. The annual growth rate of Isabela's population almost doubled between the 1980s (3.7%) and the 1990s (6.4%), a total of 1749 inhabitants being registered in the 2006 census; most of them live in the village [26].

**Figure 2:**
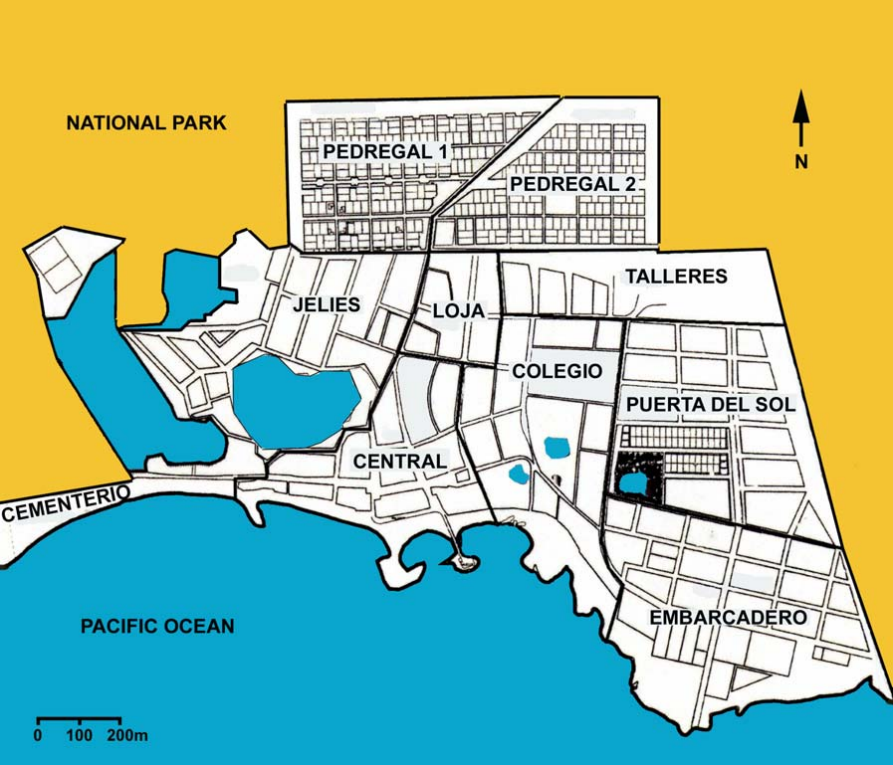
of Puerto Villamil with administrative divisions (2005) Source: Municipality of Isabela, Galápagos.

This study centered on the urban administrative territory of Southern Isabela referred to as Puerto Villamil. It covers an area of 125.2 ha and is composed of 1193 properties, grouped into 134 blocks, and forming ten neighborhoods (Figure 2).

### Sampling methodology and data collection

#### Inventory and distribution

Every property of the village was visited in order to register the presence of all the introduced species of vascular plants in the urban zone. Out of each block, five properties were randomly selected. In each of these, all the species of introduced plants were recorded and additional information to be used later in a weed risk assessment collected (see details below). The other properties were also visited to register any additional species not already encountered in the selected lots. The geographical coordinates of the lots and of new species were also recorded.

In order to determine the actual or potential invasiveness of each species, the following ecological, biological, ethno-botanical and socio-geographical aspects were recorded:

*Growth form:*

- tree-a woody plant with generally one major trunk,
- shrub-a woody plant with several stems, usually shorter than a tree,
- subshrub, a plant with some woody growth, intermediate between herb and shrub,
- herb, a plant with little or no woody growth,
- vine, a woody or non-woody plant which cannot stand freely,
- succulent, a xerophytic plant with fleshy or succulent stems and/or leaves
- aquatic

*Stage of development:* adult, juvenile or seedling**.**

*Cultivated*: planted and taken care of by man for a specific purpose.

*Not cultivated:* not planted and growing without the assistance of man.

*Presence of seedlings*: absence or presence of seedlings in the proximity of a mother plant and if present, their origin (sexual or asexual); distance from the nearby adults, abundance or surface area covered, type of substrate, humidity, light, presence of parasites, attractiveness to birds.

*Reproductive structures:* absence or presence of flowers and/or fruits and their stage of development.

*Use:* the use given to the plant by the local inhabitants: edible, ornamental, medicinal and others (for shade, wind protection, property delimitation, wood supply).

*Complementary information:* we registered land use (private housing, hotels and restaurants, vacant lots). When available, extra information was obtained from the inhabitants: common names given to the plant, date and reason of introduction of the species, information about the person who introduced it, and any specific plant behavior. This information was collected through casual conversation, focusing on any unusual species present in the garden.

*Introduction status* **:** five established categories for the introduced taxa in Galapagos were used: doubtfully native (possibly introduced); cultivated (introduced for cultivation, not naturalized); escaped (introduced for cultivation, naturalized); accidental (introduced unintentionnally, naturalized); doubtfully accidental (introduced, naturalized but not known if introduction was intentional or not).

*Naturalization assessment*: “An introduced established species, able to reproduce sexually and/or vegetatively without human assistance”: based on this definition of a naturalized species, the naturalization analysis considered the form of introduction (intentional or accidental), whether the plant was cultivated or not, the presence of sexual or/and vegetative regeneration and the presence of flowers and fruits. Combining the different criteria, three categories were established: initial, medium and complete naturalization.
